# Longer and Deeper Desaturations Are Associated With the Worsening of Mild Sleep Apnea: The Sleep Heart Health Study

**DOI:** 10.3389/fnins.2021.657126

**Published:** 2021-04-28

**Authors:** Tuomas Karhu, Sami Myllymaa, Sami Nikkonen, Diego R. Mazzotti, Juha Töyräs, Timo Leppänen

**Affiliations:** ^1^Department of Applied Physics, University of Eastern Finland, Kuopio, Finland; ^2^Diagnostic Imaging Center, Kuopio University Hospital, Kuopio, Finland; ^3^Division of Medical Informatics, Department of Internal Medicine, University of Kansas Medical Center, Kansas City, KS, United States; ^4^School of Information Technology and Electrical Engineering, The University of Queensland, Brisbane, OLD, Australia

**Keywords:** sleep apnea, intermittent hypoxemia, hypoxic burden, desaturation, progression, risk factor, oxygen saturation, disease worsening

## Abstract

**Study Objectives:**

Obesity, older age, and male sex are recognized risk factors for sleep apnea. However, it is unclear whether the severity of hypoxic burden, an essential feature of sleep apnea, is associated with the risk of sleep apnea worsening. Thus, we investigated our hypothesis that the worsening of sleep apnea is expedited in individuals with more severe desaturations.

**Methods:**

The blood oxygen saturation (SpO_2_) signals of 805 Sleep Heart Health Study participants with mild sleep apnea [5 ≤ oxygen desaturation index (ODI) < 15] were analyzed at baseline and after a mean follow-up time of 5.2 years. Linear regression analysis, adjusted for relevant covariates, was utilized to study the association between baseline SpO_2_-derived parameters and change in sleep apnea severity, determined by a change in ODI. SpO_2_-derived parameters, consisting of ODI, desaturation severity (DesSev), desaturation duration (DesDur), average desaturation area (avg. DesArea), and average desaturation duration (avg. DesDur), were standardized to enable comparisons between the parameters.

**Results:**

In the group consisting of both men and women, avg. DesDur (β = 1.594, *p* = 0.001), avg. DesArea (β = 1.316, *p* = 0.004), DesDur (β = 0.998, *p* = 0.028), and DesSev (β = 0.928, *p* = 0.040) were significantly associated with sleep apnea worsening, whereas ODI was not (β = −0.029, *p* = 0.950). In sex-stratified analysis, avg. DesDur (β = 1.987, *p* = 0.003), avg. DesArea (β = 1.502, *p* = 0.024), and DesDur (β = 1.374, *p* = 0.033) were significantly associated with sleep apnea worsening in men.

**Conclusion:**

Longer and deeper desaturations are more likely to expose a patient to the worsening of sleep apnea. This information could be useful in the planning of follow-up monitoring or lifestyle counseling in the early stage of the disease.

## Introduction

Sleep apnea is a common nocturnal breathing disorder in which breathing is interrupted numerous times during sleep. These interruptions are usually associated with transient drops in blood oxygen saturation (SpO_2_) and/or arousals from sleep. The apnea–hypopnea index (AHI) is the most widely used parameter in sleep apnea diagnostics and is derived from polysomnography (PSG) (Kapur et al., 2017). However, PSG is a labor-intensive and expensive method and experienced technicians are required to set up and monitor patients during in-laboratory PSGs or instruct patients using in-home PSG equipment. Therefore, alternative recording setups containing fewer channels have been developed (Krishnaswamy et al., 2015). The oxygen desaturation index (ODI), determined from the SpO_2_ signal, could be alternatively used as a parameter in sleep apnea screening (AASM, 1999). The ODI is a good AHI predictor due to its high correlation (Tsai et al., 1999). Furthermore, the AHI and ODI can be accurately determined from the SpO_2_ signal using neural networks (Nikkonen et al., 2019). Therefore, screening of sleep apnea and monitoring of disease progression could be based on a simple and low-cost pulse oximetry measurement.

Male sex, obesity, and older age are known risk factors for sleep apnea (Young et al., 1993; Young et al., 2004). In addition, neck circumference (NC) (Ahbab et al., 2013; Caffo et al., 2010) and the neck circumference/height ratio (NC/H) (Davies et al., 1992; Ho et al., 2016) are independent risk factors for sleep apnea. However, assessing the risk of sleep apnea progression by body mass index (BMI), NC, age, snoring, and/or upper airway structure is challenging. For example, both positive (Redline et al., 2003; Tishler et al., 2003; Lin et al., 2015) and negative (Sforza et al., 1994; Pendlebury et al., 1997; Berger et al., 2009) results on whether the BMI is a factor for sleep apnea worsening have been reported. Similarly, there are conflicting results about whether higher baseline ODI values are associated with an expedited worsening of sleep apnea (Sforza et al., 1994; Lin et al., 2015).

Patients with mild sleep apnea are not systematically treated, especially if symptomless, despite being the most prone to the worsening of the disease (Sforza et al., 1994; Berger et al., 2009). Moreover, even though the severities of individual respiratory events vary within mild sleep apnea patients (Kulkas et al., 2013a, b) and are generally associated more strongly with severe health consequences than the AHI (Muraja-Murro et al., 2013, 2014), the severity of individual events are ignored in current sleep apnea diagnostics. To address these shortcomings, we have introduced novel SpO_2_-derived parameters (Kulkas et al., 2013a) to quantify the severity of the hypoxic burden and physiological stress experienced by a patient. An elevated hypoxic burden has been associated with several sleep apnea-related comorbidities (Stone et al., 2016; Azarbarzin et al., 2019, 2020), while the AHI and ODI have not. Therefore, novel parameters considering the severities of individual desaturation events could describe the true severity of sleep apnea better than the AHI and ODI (Otero et al., 2012; Kulkas et al., 2013a). However, it is unknown whether mild sleep apnea patients with deep and long desaturations have an elevated risk of expedited worsening of the disease. We hypothesize that mild sleep apnea patients with severe desaturations at baseline experience an expedited worsening of sleep apnea severity. To investigate this, we evaluated the effect of baseline hypoxemia markers on the progression of mild sleep apnea in 805 Sleep Heart Health Study participants.

## Materials and Methods

### Dataset

The Sleep Heart Health Study (SHHS) is a multicenter cohort study implemented by the National Heart, Lung, and Blood Institute to determine the consequences of sleep-disordered breathing, such as cardiovascular diseases (CVD). The SHHS dataset is available through the National Sleep Research Resource (Quan et al., 1997; Zhang et al., 2018; The National Sleep Research Resource, 2021). Participants were recruited from nine existing parent cohort studies and provided informed consent for data collection. Successful baseline PSG examination was performed for 6,441 participants between 1995 and 1998, who met the following inclusion criteria: (1) age ≥40 years; (2) no history of sleep apnea treatment; (3) no tracheostomy; and (4) no current home oxygen therapy. The follow-up PSG was performed between 2001 and 2003 for 3,295 participants who were not treated for sleep apnea with positive continuous airway pressure, oral device, or oxygen therapy 3 months prior to the follow-up PSG. Due to the sovereignty issues with one of the parent studies (Strong Heart Study), data from approximately 600 participants are not available. Moreover, due to data corruption over time, data have been lost from a few participants. Therefore, 5,793 baseline and 2,651 follow-up PSGs are available; out of these, 2,647 participants have both recordings available. More details on the SHHS dataset are available elsewhere (Quan et al., 1997; Redline et al., 1998; Dean et al., 2016).

### Polysomnography and Covariates

In-home PSGs were performed with Compumedics P-series portable monitors (Abbotsford, Australia) (Quan et al., 1997; Redline et al., 1998). The finger pulse oximeters (Nonin XPOD model 3011, Minneapolis, MN, United States) were used to record SpO_2_ with a 1-Hz sampling frequency. Mercury gauge sensors were used to record the body position during sleep. Total sleep time was determined based on 30-s epochs in which the sleep stage was scored as non-rapid eye movement sleep (N1, N2, or N3) or rapid eye movement sleep (REM).

Each PSG recording was supplemented with a sleep habits questionnaire, medical history, medication usage, blood pressure, and anthropometric measurements. NC was measured just below the laryngeal prominence. The existence of hypertension was defined if the systolic blood pressure was ≥140 mmHg, diastolic blood pressure was ≥90 mmHg, or medication for hypertension was in use. At the medical history interview, history of CVD, consisting of myocardial infarction, heart failure, stroke, coronary angioplasty, and coronary artery bypass graft, was inquired. In addition, the existence of diabetes was defined based on self-reported diabetes status and usage of insulin or oral hypoglycemic agents.

### Oxygen Desaturation Parameters

Oxygen saturation signals were reanalyzed due to known issues of data corruption and loss of scored event data in the SHHS (The National Sleep Research Resource, 2021). To improve data consistency, desaturations were automatically re-scored using Noxturnal software (version 5.1.19824, Nox Medical, Reykjavík, Iceland). The scoring criteria for desaturations were: (1) minimum of 3% drop in the SpO_2_ signal; (2) minimum event duration of 3 s; (3) maximum plateau duration of 45 s; and (4) values lower than 50% were considered as artifacts (no desaturations were scored in these parts of the signal). The maximum plateau duration denotes the maximum period within the desaturation event during which the SpO_2_ signal values do not change. If this period is exceeded, the end point of the desaturation is determined to be the starting point of the plateau. It was observed that the software started automatic event scorings systematically one data point too early, and thus, this was corrected in the parameter calculations. To validate the accuracy of the automatic scorings, 30 SpO_2_ signals were randomly selected from the available SHHS dataset of 8,444 recordings and scored manually. Correlations and Bland–Altman plot agreements between manual and automatic scorings of the desaturation events were calculated. In addition to the ODI, novel SpO_2_ signal-based parameters consisting of desaturation severity (DesSev), desaturation duration (DesDur), average desaturation duration (avg. DesDur), and average desaturation area (avg. DesArea) were calculated (Table 1; see Kulkas et al., 2013a). These parameters describe the hypoxic burden by taking into account the duration and depth of the desaturation events.

**TABLE 1:** Descriptions and formulas of desaturation parameters.

Parameter	Description	Formula
ODI (1/h)	Average number of desaturation events per hour of sleep	$\frac{n_{\mathrm{desat}\mathrm{events}}}{\text{TST}}$
DesDur*_*i*_* (s)	Desaturation duration of a single desaturation event	*t_2-t_1*
DesArea*_*i*_* (s%)	Desaturation area of a single desaturation event	$\int_{t_{1}}^{t_{2}}{\text{SpO}}_{2}\left(t\right)dt$
DesSev (%)	Total desaturation area normalized with total sleep time	$\frac{\sum_{i}{\text{DesArea}}_{i}}{\text{TST}}$
DesDur (%)	Total desaturation duration normalized with total sleep time	$\frac{\sum_{i}{\text{DesDur}}_{i}}{\text{TST}}\times 100\%$
Avg. DesArea (s%)	Average area of individual desaturation events	$\frac{\sum_{i}{\text{DesArea}}_{i}}{n_{\mathrm{desat}\mathrm{events}}}$
Avg. DesDur (s)	Average duration of individual desaturation events	$\frac{\sum_{i}{\text{DesDur}}_{i}}{n_{\mathrm{desat}\mathrm{events}}}$

*n_desat events_ is the number of desaturation events and TST is the total sleep time. t_1_ and t_2_ denote the start and end time points of a single desaturation event, respectively, in the SpO_2_ signal. ODI, oxygen desaturation index; DesSev, desaturation severity parameter; DesDur, desaturation duration parameter; avg. DesArea, average area of individual desaturation events; avg. DesDur, average duration of individual desaturation events.*

### Sleep Apnea Severity Classification

The severity of sleep apnea was determined based on the ODI 4% criterion for several reasons. First, only desaturation events fulfilling the minimum transient drop of 4% were included in the analysis as the 4% criterion was considered more reliable than the 3% criterion, as the desaturations were scored automatically and separately from respiratory events. Second, the ODI is known to be a good predictor of AHI (Tsai et al., 1999; Chung et al., 2012; Fabius et al., 2019). Third, originally apneas and hypopneas were scored based on the thermistor, respiratory belts, or some combination of them (The National Sleep Research Resource, 2021). Therefore, scored respiratory events are not in line with the current standards. In addition, the hypoxic burden is an important feature of sleep apnea pathophysiology (Dempsey et al., 2010), and thus, the usage of ODI in the assessment of sleep apnea severity can be justified. In the present study, the term “progression” refers to a change in ODI (either an increase or a decrease) between the two PSG recordings, whereas “worsening” refers to an increase in ODI.

Out of the 2,647 participants with both PSG recordings, 832 had mild sleep apnea (5 ≤ ODI < 15) at baseline, from which 27 were excluded due to the missing covariate data. Therefore, 805 (441 men and 364 women) participants were included for further analyses (Table 2). The results utilizing the ODI 3% criterion are presented in Supplementary Tables 1–5 and Supplementary Figure 1.

**TABLE 2:** Demographic, anthropometric, and desaturation parameter values for men and women participants with mild sleep apnea at baseline and after a mean follow-up time of 5.2 years.

Parameter	Men			Women		
	Baseline	Follow-up	Change during the follow-up	Baseline	Follow-up	Change during the follow-up
Number of patients, *n*		441			364	
Follow-up time (years)		5.2 (0.3)			5.2 (0.2)	
Age (years)	64.1 (9.7)	69.4 (9.5)^a^	5.2 (0.6)	65.0 (10.3)	70.1 (10.2)^a^	5.1 (0.6)^b^
BMI (kg/m^2^)	28.9 (3.7)	29.0 (4.0)	0.1 (1.7)	29.7 (5.7)	29.5 (5.9)	−0.2 (2.4)
TST (h)	6.0 (1.0)	6.0 (1.2)	0.0 (1.2)	6.1 (1.1)	6.2 (1.2)	0.1 (1.3)
Supine time (%)	23.3 (0.0–52.9)	25.0 (8.0–55.0)	1.9 (38.8)	32.7 (1.1–63.6)^b^	36.0 (12.3–60.8)	2.6 (44.0)
NC (cm)	40.9 (3.0)	40.7 (2.9)	−0.2 (2.3)	35.8 (2.9)^b^	35.7 (3.1)	−0.1 (2.2)
NC/H (%)	23.5 (1.8)	23.6 (1.8)	0.1 (1.4)	22.3 (1.9)^b^	22.5 (2.0)^a^	0.2 (1.4)
REM (%)	19.6 (5.9)	19.6 (6.6)	0.0 (7.8)	19.7 (6.9)	20.1 (6.7)	0.4 (8.4)
Hypertension, *n* (%)	219 (49.7)	252 (57.1)		203 (55.8)	216 (59.3)	
Diabetes, *n* (%)	42 (9.5)	n.a.		29 (8.0)	n.a.	
CVD, *n* (%)	74 (16.8)	107 (24.3)		25 (6.9)^b^	43 (11.8)	
ODI (1/h)	8.7 (6.8–11.5)	16.4 (10.3–25.1)^a^	10.6 (14.5)	8.0 (6.3–10.7)^b^	13.0 (7.9–20.4)^a^	7.4 (11.3)^b^
DesSev (%)	0.27 (0.11)	0.58 (0.55)^a^	0.31 (0.52)	0.24 (0.11)^b^	0.44 (0.34)^a^	0.19 (0.31)^b^
DesDur (%)	8.3 (2.9)	17.1 (12.3)^a^	8.8 (11.6)	7.4 (2.8)^b^	13.4 (9.1)^a^	6.0 (8.6)^b^
Avg. DesArea (s%)	107.2 (29.6)	103.2 (30.0)^a^	−3.9 (32.0)	100.9 (29.9)^b^	96.7 (28.2)^a^	−4.1 (27.2)
Avg. DesDur (s)	31.8 (6.8)	31.5 (6.8)	−0.3 (7.4)	29.9 (6.9)^b^	30.0 (6.6)	0.1 (7.1)

*Data are presented as means and standard deviations for normally distributed variables, as medians and interquartile ranges for non-normally distributed variables, and as n and percentages for categorical variables. BMI, body mass index; TST, total sleep time; NC, neck circumference; NC/H, neck circumference/height ratio; REM, rapid eye movement sleep; CVD, cardiovascular disease (consisting of heart failure, stroke, myocardial infarction, coronary artery bypass graft, and coronary angioplasty); ODI, oxygen desaturation index; DesSev, desaturation severity parameter; DesDur, desaturation duration parameter; avg. DesArea, average area of individual desaturation events; avg. DesDur, average duration of individual desaturation events; n.a., not available. ^a^Statistical significance (p < 0.05) between baseline and follow-up measurements was determined with Wilcoxon signed-rank test. ^b^Mann–Whitney U and Chi-squared tests were used to compare the baseline parameter values for continuous and categorical variables, respectively, and changes in the parameter values during the follow-up between the sexes.*

### Statistical Analysis

The statistical significance of the differences in the demographic and desaturation parameters between the baseline and follow-up were evaluated within men and women using the Wilcoxon signed-rank test, and between men and women with the Mann–Whitney *U* test and Chi-squared test for continuous and categorical variables, respectively. Linear regression was used to investigate the association between the baseline desaturation parameters and the progression of mild sleep apnea with and without covariate adjustment. Change in the ODI between the PSG recordings was used as a continuous dependent variable. Baseline BMI, change in BMI during the follow-up, age, NC/H, the existence of hypertension, diabetes, and CVD, percentage of time slept in the supine position, percentage of time slept in REM, change in the time slept in REM between the PSGs, and follow-up time were used as covariates in the adjusted models. Desaturation parameters at baseline were standardized to enable comparisons between parameters. Thus, regression coefficients (β values) correspond to the expedited increase in ODI between the PSG recordings that were associated with a one standard deviation (SD) change in the desaturation parameter values at baseline. In addition, we investigated whether the desaturation parameter values at baseline differed between the participants whose sleep apnea severity remained in the healthy-to-mild state (i.e., ODI < 15) and the participants whose disease worsened to moderate (15 ≤ ODI < 30) or severe (ODI ≥ 30) sleep apnea during the follow-up. Finally, to address the possibility of selection bias, we investigated whether there were differences in the baseline parameter values between the participants with mild sleep apnea who underwent only baseline PSG and those with both PSGs. Analyses were conducted in MATLAB^®^, (version 2018b, MathWorks, Natick, MA, United States). To address the multiple comparisons, due to five investigated desaturation parameters, a Bonferroni-corrected *p*-value threshold of <0.01 was used to indicate statistical significance, whereas *p*-values < 0.05 were considered as nominal evidence.

## Results

During the follow-up, the ODI, DesSev, and DesDur values increased for both sexes (*p* < 0.001), whereas avg. DesArea decreased (*p* < 0.001 for men, *p* = 0.004 for women) (Table 2). The increase in ODI (*p* = 0.002), DesSev (*p* = 0.002), and DesDur (*p* < 0.001) was greater in men than in women.

Linear regression analyses revealed that the baseline ODI was not associated with the worsening of mild sleep apnea either in the unadjusted or in the adjusted model (Table 3). However, in men and in the group consisting of both sexes, all novel desaturation parameters were significantly (*p* < 0.05) associated with sleep apnea worsening in the unadjusted models. In the covariate-adjusted models for the group consisting of both sexes, avg. DesArea (*p* = 0.001) and avg. DesDur (*p* = 0.004) were significantly associated with sleep apnea worsening by fulfilling the Bonferroni-corrected threshold, while DesSev (*p* = 0.040) and DesDur (*p* = 0.028) reached the limit of nominal significance. Moreover, in men, avg. DesDur was associated with sleep apnea worsening at the Bonferroni-corrected threshold (*p* = 0.003), while avg. DesArea (*p* = 0.024) and DesDur (*p* = 0.033) reached nominal association. Overall, in men and in the group consisting of both sexes, a one SD unit increase in avg. DesDur resulted in the greatest expedited increase in ODI during the follow-up (i.e., largest β values), followed by avg. DesArea.

**TABLE 3:** Linear regression analyses for the estimation of mild sleep apnea progression based on the desaturation parameter values at the baseline.

	Unadjusted			Adjusted^a^		
	β	SD error	*p*-value	β	SD error	*p*-value
**ODI (1/h)**						

Men	0.705	0.691	0.308	0.021	0.646	0.974
Women	−0.079	0.593	0.894	−0.246	0.614	0.688
Both	0.519	0.466	0.266	−0.029	0.458	0.950

**DesSev (%)**						

Men	1.652	0.687	0.017	1.169	0.644	0.070
Women	0.616	0.592	0.299	0.447	0.595	0.453
Both	1.378	0.464	0.003	0.928	0.450	0.040

**DesDur (%)**						

Men	1.948	0.685	0.005	1.374	0.644	0.033
Women	0.473	0.592	0.425	0.278	0.599	0.643
Both	1.518	0.463	0.001	0.998	0.453	0.028

**Avg. DesArea (s%)**						

Men	1.506	0.688	0.029	1.502	0.661	0.024
Women	0.988	0.590	0.095	0.951	0.593	0.110
Both	1.428	0.464	0.002	1.316	0.454	0.004

**Avg. DesDur (s)**						

Men	1.914	0.685	0.005	1.987	0.661	0.003
Women	0.986	0.590	0.096	0.888	0.603	0.142
Both	1.696	0.463	<0.001	1.594	0.458	0.001

*β values correspond to the expedited increase in ODI between the PSG recordings that is associated with a one standard deviation change in the desaturation parameter values at the baseline. Standard deviations for men, women, and the group consisting of both sexes were respectively: for ODI = 2.8, 2.7, and 2.7; for DesSev = 0.11, 0.11, and 0.12; for DesDur = 2.9, 2.8, and 2.9; for avg. DesArea = 29.6, 29.9, and 29.9; and for avg. DesDur = 6.8, 6.9, and 6.9. ODI, oxygen desaturation index; DesSev, desaturation severity parameter; DesDur, desaturation duration parameter; avg. DesArea, average area of individual desaturation events; avg. DesDur, average duration of individual desaturation events. ^a^Adjusted for age, body mass index, change in body mass index during the follow-up, neck circumference/height ratio, the existence of hypertension, diabetes, and cardiovascular diseases (consisting of heart failure, stroke, myocardial infarction, coronary artery bypass graft, and coronary angioplasty), percentage of time slept in the supine position, percentage of time slept in rapid eye movement sleep, change in rapid eye movement sleep between the polysomnography recordings, and follow-up time.*

Men and women whose mild sleep apnea worsened to moderate sleep apnea during the follow-up had significantly higher ODI (*p* < 0.001 for men, *p* = 0.008 for women), DesSev (*p* < 0.001 for men, *p* < 0.001 for women), and DesDur (*p* < 0.001 for men, *p* < 0.001 for women) at baseline compared to the participants who remained in the healthy-to-mild state (Table 4). Similar findings were observed in men (*p* < 0.001 for ODI, DesSev, and DesDur) and women (*p* < 0.001 for ODI, *p* = 0.001 for DesSev, and *p* = 0.001 for DesDur) whose mild sleep apnea worsened to severe sleep apnea during the follow-up. In addition, avg. DesArea (*p* < 0.001) and avg. DesDur (*p* = 0.021) were significantly higher at baseline in women whose disease worsened to moderate sleep apnea. The only statistically significant difference between the participants who worsened to moderate sleep apnea and those who worsened to severe sleep apnea was observed in ODI (*p* = 0.039) in the group consisting of both sexes.

**TABLE 4:** Desaturation parameter values at baseline in participants whose sleep apnea severity remained in the healthy-to-mild state (ODI < 15) and in those whose disease worsened to moderate (15 ≤ ODI < 30) or severe (ODI ≥ 30) sleep apnea during the follow-up.

	ODI < 15	15 ≤ ODI < 30	ODI ≥ 30
**ODI (1/h)**			

Men	7.9 (6.3–10.3)	9.7 (7.1–12.0)^a^	9.9 (7.5–12.2)^a^
Women	7.5 (6.1–9.8)	8.5 (6.4–10.9)^a^	9.8 (7.6–12.0)^a^
Both	7.8 (6.2–10.0)	9.2 (6.8–11.7)^a^	9.9 (7.5–12.2)^ab^

**DesSev (%)**			

Men	0.24 (0.11)	0.29 (0.11)^a^	0.31 (0.13)^a^
Women	0.22 (0.10)	0.27 (0.12)^a^	0.29 (0.13)^a^
Both	0.23 (0.10)	0.28 (0.11)^a^	0.30 (0.13)^a^

**DesDur (%)**			

Men	7.5 (2.7)	8.8 (2.8)^a^	9.3 (3.1)^a^
Women	6.8 (2.7)	8.0 (2.8)^a^	8.6 (3.0)^a^
Both	7.2 (2.7)	8.4 (2.8)^a^	9.0 (3.1)^a^

**Avg. DesArea (s%)**			

Men	104.0 (28.1)	110.7 (31.7)	108.2 (28.2)
Women	96.5 (28.7)	108.4 (31.5)^a^	101.5 (26.9)
Both	100.2 (28.6)	109.7 (31.6)^a^	106.1 (27.9)^a^

**Avg. DesDur (s)**			

Men	31.3 (6.9)	32.4 (6.9)	32.2 (6.5)
Women	29.1 (6.8)	31.3 (7.4)^a^	29.9 (5.0)
Both	30.2 (6.9)	31.9 (7.1)^a^	31.5 (6.1)

*Data are presented as means and standard deviations for normally distributed variables and as medians and interquartile ranges for non-normally distributed variables. Mann–Whitney U test was used to determine the statistical significance (p < 0.05) between the participants who remained in the healthy-to-mild state (n_Men_ = 206, n_Women_ = 209) and those who worsened to moderate (n_Men_ = 158, n_Women_ = 119) or severe (n_Men_ = 77, n_Women_ = 36) sleep apnea (^a^) and between participants who worsened to moderate sleep apnea and those who worsened to severe sleep apnea (^b^). ODI, oxygen desaturation index; DesSev, desaturation severity parameter; DesDur, desaturation duration parameter; avg. DesArea, average area of individual desaturation events; avg. DesDur, average duration of individual desaturation events.*

No statistically significant differences in the baseline desaturation parameters were observed between the participants with mild sleep apnea who underwent only baseline PSG and those who underwent both PSGs (Table 5).

**TABLE 5:** Comparison between participants with mild sleep apnea at baseline who participated only to the baseline PSG and those who participated also to the follow-up PSG.

	Participants with only baseline PSG	Participants with both PSGs	*p*-value
*n* (men, women)	871 (458, 413)	805 (441, 364)	
Age (years)	67.0 (10.7)	64.5 (10.0)	<0.001
BMI (kg/m^2^)	29.0 (5.0)	29.2 (4.7)	0.083
Supine time (%)	22.9 (0.0–58.9)	26.6 (0.1–57.2)	0.421
NC (cm)	38.7 (3.9)	38.6 (3.9)	0.604
NC/H (%)	23.1 (1.9)	22.9 (1.9)	0.177
REM (%)	18.6 (7.1)	19.7 (6.4)	0.015
Hypertension, *n* (%)	557 (63.9)	422 (52.4)	<0.001
Diabetes, *n* (%)	91 (10.4)	71 (8.8)	0.260
CVD, *n* (%)	159 (18.3)	99 (12.3)	0.001
ODI (1/h)	8.6 (6.6–11.0)	8.4 (6.5–11.0)	0.636
DesSev (%)	0.26 (0.12)	0.26 (0.12)	0.739
DesDur (%)	7.9 (3.0)	7.9 (2.9)	0.859
Avg. DesArea (s%)	104.3 (32.7)	104.3 (29.9)	0.765
Avg. DesDur (s)	30.8 (7.2)	31.0 (6.9)	0.406

*Values are presented as means and standard deviations for normally distributed parameters, as medians and interquartile ranges for non-normally distributed parameters, and as n and percentages for categorical variables. Statistical comparison of the observed differences between the populations was investigated with Mann–Whitney U test for continuous variables and with Chi-squared test for categorical variables. BMI, body mass index; NC, neck circumference; NC/H, neck circumference/height ratio; REM, rapid eye movement sleep; CVD, cardiovascular diseases (consisting of myocardial infarction, heart failure, stroke, coronary angioplasty, and coronary artery bypass graft); ODI, oxygen desaturation index; DesSev, desaturation severity parameter; DesDur, desaturation duration parameter; avg. DesArea, average area of individual desaturation events; avg. DesDur, average duration of individual desaturation events.*

**FIGURE 1:**
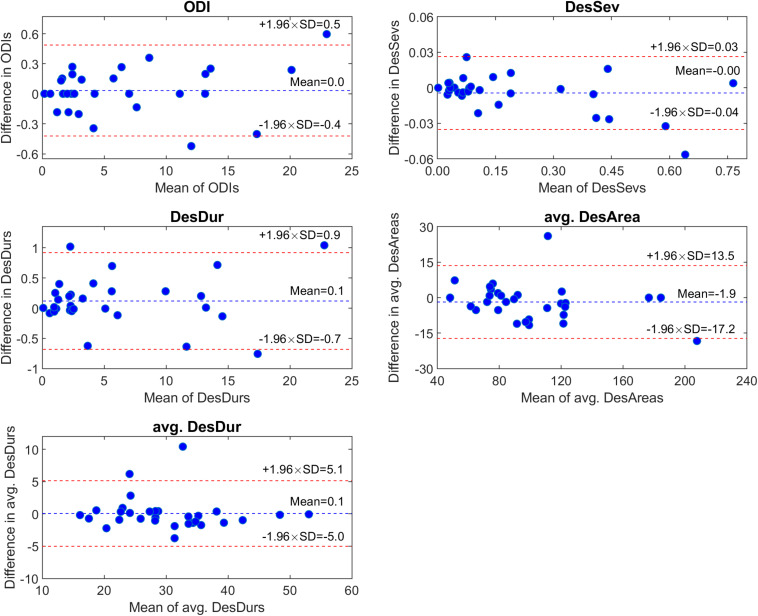
Comparison of automatically and manually scored desaturation parameter values of 30 randomly selected oxygen saturation signals using Bland-Altman plots. ODI, oxygen desaturation index; DesSev, desaturation severity parameter; DesDur, desaturation duration parameter; avg. DesArea, average area of individual desaturation events; avg. DesDur, average duration of individual desaturation events.

The automatic scoring of the desaturation events was very well in line with the manual scoring. For all five desaturation parameters, the correlations between the manual and automatic scorings were excellent (ρ ≥ 0.94), the median differences in the parameter values were minimal (Table 6), and agreements in the parameter values were strong (Figure 1).

**TABLE 6:** Automatically and manually scored oxygen saturation signal-derived parameters were highly similar and strongly correlated.

Parameter	Automatic scorings	Manual scorings	Difference	Spearman’s correlation
ODI (1/h)	3.6 (2.0–11.1)	3.7 (2.0–11.1)	0.0 (0.0–0.2)	1.00
DesSev (%)	0.09 (0.05–0.32)	0.08 (0.06–0.32)	0.00 (−0.01–0.00)	0.98
DesDur (%)	3.1 (1.6–10.1)	2.9 (1.2–9.8)	0.0 (0.0–0.3)	0.98
avg. DesArea (s%)	90.6 (76.3–118.6)	94.2 (73.1–121.1)	−1.8 (−5.3–1.1)	0.95
avg. DesDur (s)	28.7 (24.2–34.8)	28.5 (22.8–35.3)	−0.4 (−1.2–0.4)	0.94

*For this analysis, 30 oxygen saturation signals were randomly selected from the Sleep Heart Health Study dataset of 8,444 available recordings. Values are presented as medians (interquartile range). Statistical significance (p < 0.05) of the observed difference between the scorings was investigated with Wilcoxon signed-rank test (no statistically significant differences were observed). ODI, oxygen desaturation index; DesSev, desaturation severity parameter; DesDur, desaturation duration parameter; avg. DesArea, average area of individual desaturation events; avg. DesDur, average duration of individual desaturation events.*

## Discussion

In this study, we investigated whether the desaturation parameters at baseline were associated with the worsening of mild sleep apnea. We provide novel evidence showing that especially avg. DesDur and avg. DesAreas are associated with the expedited worsening of sleep apnea. Notably, the baseline ODI values did not appear to be associated with the worsening of mild sleep apnea. These findings suggest that a detailed analysis of the oxygen desaturation signal that considers the morphology of the desaturation events is relevant in the risk assessment of sleep apnea progression. More importantly, this study focused on patients with mild sleep apnea as these patients are not systematically treated especially when symptomless. Therefore, our results implicate that mild sleep apnea patients with deeper and longer desaturation events might benefit from regular follow-up monitoring.

Previously, Lin et al. (2015) demonstrated that baseline ODI is a significant predictor of the worsening of sleep apnea, which is partially contradictory to our findings. On one hand, it represents the same biological concept where increased nocturnal hypoxemia is a predictor of disease worsening. However, based on the present results, the ODI alone might not be a robust marker for assessing the progression of mild sleep apnea; a more detailed morphological assessment of individual desaturation events could provide more accurate estimates. Furthermore, the opposing findings could be partly explained by differences in the study populations and the lengths of the follow-up periods. Our study population size is significantly larger than that in the study by Lin et al. (*n* = 805 vs. *n* = 50), and we had a longer follow-up period (5.2 vs. 3 years). In addition, their study population consisted of patients who were suffering from more severe sleep apnea at baseline (ODI mean ± SD = 20.8 ± 13.4), whereas we focused only on patients with mild sleep apnea. Nevertheless, the present results are consistent with other previously reported findings (Sforza et al., 1994) with a similar follow-up period (5.7 years), but with a small population (*n* = 32) of patients with more severe sleep apnea (mean AHI = 52.2 at baseline).

Moreover, it has been shown that AHI does not change over time (mean follow-up period of 5.1 years) in severe sleep apnea patients, and the ones whose AHI increased have initially mild or moderate disease (Berger et al., 2009). It was suggested (Berger et al., 2009) that this is due to the ceiling effect of sleep apnea. Another explaining factor could be the “regression toward the mean” phenomenon, where initially extreme AHI values get closer to the mean at the follow-up measurement, and *vice versa*. Therefore, it seems that it is highly dependent on the severity of sleep apnea whether baseline ODI or AHI values can be used in the risk assessment of sleep apnea progression; thus, the generalization of our findings should be done with caution.

We observed that mild sleep apnea patients with longer and deeper desaturations experience an expedited worsening of the disease. However, in the sex-stratified analyses, significant findings were observed in men when the ODI 4% criterion was used, whereas the associations were stronger in women with the ODI 3% criterion (Supplementary Material). In addition, we noted that men had more severe desaturations than women, which is supported by previous studies (Ware et al., 2000; Schwartz et al., 2008; Peppard et al., 2009). Therefore, it could be speculated that the 4% criterion might be too strict to assess the progression of sleep apnea severity in women. Furthermore, the ODI values increased, while the avg. DesArea decreased for both sexes during the follow-up. Thus, our findings suggest that individuals whose desaturation events are more severe at baseline develop more of these less severe events. Azarbarzin et al. have also shown that the severity of hypoxic burden predicts cardiovascular mortality (Azarbarzin et al., 2019) and incident heart failure (Azarbarzin et al., 2020). Therefore, the nocturnal hypoxic burden seems to play a potential prognostic role in the worsening of sleep apnea and the development of related comorbidities.

The present study has limitations. First, the desaturations were autoscored using a commercial software without further manual adjustment by specialists. However, the correlations and agreements between the subset of manual and automatic scorings were excellent and the median differences minimal (Table 6 and Figure 1). Therefore, we were convinced that the used automatic desaturation scoring methods can be assumed to be valid. New scoring was required since part of the manually scored desaturation events had been lost due to the SHHS data corruption over time (The National Sleep Research Resource, 2021). Moreover, there are no current standardized criteria for scoring desaturation events, in addition to a minimum transient drop of 3 or 4% in the SpO_2_ signal. For example, the minimum or the maximum durations of the desaturation events are not specified in the rules of the American Academy of Sleep Medicine (AASM), unlike in the case of hypopneas and apneas (Berry et al., 2017). Furthermore, no instructions for the maximum duration of the plateau in the middle of the event exist. In this study, a minimum event duration was set to 3 s and a maximum plateau duration set to 45 s based on visual inspection in which this criterion was observed to be appropriate. However, no fine-tuning to obtain an optimized desaturation scoring criterion was performed. With a shorter plateau length, some of the events might not have filled the minimum rule of transient drop in the SpO_2_ signal, or one longer event might have been split into multiple shorter events. In contrast, with longer plateau criteria, short events could fuse into a longer one. All these aspects affect the number (ODI) and severity (novel parameters) of the events and, therefore, the determined parameter values. Furthermore, we decided to use the 4% criterion for our primary analysis as, without associating desaturation events to the respiratory events, the 3% criterion was assumed to be too sensitive.

Apnea–hypopnea index was not used for the severity categorization in this study because the scoring criteria have changed since the apneas and hypopneas were originally scored in the late 1990s and early 2000s. The biggest difference is in the channels used for hypopnea scoring. At the time the recordings were conducted, hypopneas could have been scored based on signals from the thermistor, respiratory belts around the thorax or abdominal region, or some combination of them (The National Sleep Research Resource, 2021). The current AASM recommendation for apnea scoring is an oronasal thermal airflow sensor, while a nasal pressure transducer is recommended for hypopnea scoring (Berry et al., 2017). Moreover, in addition to the desaturation events, the Noxturnal software is capable of scoring apneas and hypopneas automatically. However, the accuracy of the detection of hypopnea and apnea events without manual adjustment was found to be insufficient, and manual re-scoring of the massive SHHS dataset was not feasible. Furthermore, the oximetry used in the SHHS data acquisition differs from the current clinical recommendations. For example, the oximetry used in the SHHS utilized a sampling frequency of 1 Hz, while the current minimum recommendation for routine clinical recordings is 10 Hz (Berry et al., 2017). However, the use of a sampling frequency of 1 Hz can be considered sufficient as it has been shown not to affect the accuracy to detect sleep apnea (Nigro et al., 2011).

Using the ODI to characterize the severity of OSA has certain limitations. As the SpO_2_ is not a direct measure of breathing, the ODI cannot be used as a direct measure of the frequency of respiratory events. In addition, the ODI cannot distinguish between obstructive and central respiratory events. Furthermore, no standardized criteria exist to score desaturations, as discussed above. The ODI can also potentially underestimate the AHI, as hypopneas can be scored with an association to desaturation or arousal (Berry et al., 2017). In addition, apneas can be scored without desaturation or arousal, or multiple respiratory events can be associated with a single desaturation. However, using the ODI for the severity categorization is adequate as it has been shown that only 6.3% of apneas are not associated with desaturations and 4.7% of desaturations are not associated with respiratory events (Fabius et al., 2019). Thus, misclassification due to unmatched events can be assumed to be minor.

Another limitation is the potential influence of night-to-night variability in the assessment of sleep apnea severity. It has been shown that there is significant intra-patient variability in the AHI between two consecutive nights (Roeder et al., 2020). However, no such night-to-night variability was observed at the group level (Roeder et al., 2020). Therefore, it is likely that such variations are averaged out in our relatively large study population. Finally, the study population was relatively old and could be enriched with participants with CVD, due to the study design of the SHHS, thus potentially causing selection bias.

Our present findings give a new perspective on the risk assessment of whether mild sleep apnea will worsen over time. The consideration of individual desaturation event severities could be an additional tool in the planning of regular follow-up monitoring, initiation of treatment, or lifestyle counseling. With these preventive actions, sleep apnea worsening could be slowed or prevented earlier. Adequate management of mild sleep apnea patients with severe desaturation events could further lower the risks of sleep apnea-related comorbidities and generally improve the quality of life. However, regular monitoring of all mild sleep apnea patients would be complicated and expensive with the current diagnostic methods (i.e., polysomnography). In addition, treating a large number of mild sleep apnea patients would be costly while not providing significant benefits for many of the patients, thus being a waste of resources. Therefore, using novel SpO_2_-derived parameters in the risk assessment of sleep apnea worsening and planning of the follow-up monitoring and interventions could be a practical approach. This would allow cost-efficient regular monitoring of sleep apnea using a simple pulse oximeter often included, e.g., in many consumer-grade wearable devices. This could also enable reducing the effect of night-to-night variability on sleep apnea severity estimation (Stöberl et al., 2017; Roeder et al., 2020), allowing more reliable diagnosis and prognosis.

## Conclusion

The present results indicate that, in the risk assessment of mild sleep apnea worsening, the severity of the desaturation events is more useful than the exact number of the events. Based on the present findings, sleep apnea can be understood as a progressive disease, and many of the mild patients develop more severe disease in 5 years.

## Data Availability Statement

Publicly available datasets were analyzed in this study. This data can be found here: National Sleep Research Resource: https://sleepdata.org/datasets/shhs/. Access to the full Sleep Heart Health Study data was granted by the National Sleep Research Resource as a part of Mazzotti’s proposal (agreement #2731).

## Ethics Statement

Each patient/participant provided written informed consent and the study protocol was reviewed and approved by the institutional review boards of each participating site of the Sleep Heart Health Study (SHHS). Participating institutions in the Sleep Heart Health Study are (https://sleepdata.org/datasets/shhs/pages/full-description.md): Boston University, Case Western Reserve University, Johns Hopkins University, Missouri Breaks Research, Inc. New York University Medical Center, University of Arizona, University of California at Davis, University of Minnesota – Clinical and, Translational Science Institute, University of Washington.

## Author Contributions

TK contributed to the study design, data analysis, and interpretation, and wrote the manuscript. SM contributed to the study design and writing of the manuscript. SN contributed to the writing of the manuscript and data analysis. DM contributed to data interpretation and writing of the manuscript. JT and TL contributed to the study design, data interpretation, and writing of the manuscript. All authors contributed to the article and approved the submitted version.

## Conflict of Interest

The authors declare that the research was conducted in the absence of any commercial or financial relationships that could be construed as a potential conflict of interest.
