# Supplementary material for: Longer and Deeper Desaturations Are Associated With the Worsening of Mild Sleep Apnea: The Sleep Heart Health Study
Source: Front Neurosci. 2021 Apr 28;15:657126. doi: 10.3389/fnins.2021.657126 (PMC8113677; doi:10.3389/fnins.2021.657126)
Supplement: Supplementary file 1 [file Table_1.docx]

Supplementary Material

# Supplementary results

Out of 2647 participants with both PSG recordings, 1013 had mild sleep apnea (5≤ODI3%<15) at the baseline from which 31 were excluded due to the missing covariate data. Therefore, 982 (393 men, 589 women) participants were included for further analyses (Supplementary Table 1).

During the follow-up, ODI, DesSev, and DesDur values increased for both sexes (*p*<0.001) (Supplementary Table 1). Moreover, the average DesArea increased in men (*p*=0.027) and in women (*p*<0.001), whereas the average DesDur decreased in men (*p*=0.018). However, in the linear regression analyses, baseline ODI3% was not associated with the worsening of mild sleep apnea in either unadjusted or adjusted models in men, women, or in the group consisting of both sexes (Supplementary Table 2). However, in the covariate-adjusted analyses, the average DesArea (*p*<0.001) and average DesDur (*p*=0.002) were associated with the worsening of mild sleep apnea in the group consisting of both sexes at the Bonferroni-corrected threshold, while DesSev reached nominal association (*p*=0.021). In sex-stratified analyses, the average DesDur in women (*p*=0.007) and DesSev in men (*p*=0.008) had a significant association with the sleep apnea worsening at the Bonferroni-corrected threshold. Moreover, the average DesArea in men (*p*=0.037) and women (*p*=0.019) showed evidence of nominal association.

Men and women whose mild sleep apnea worsened to moderate sleep apnea at the follow-up, had significantly higher ODI, DesSev, and DesDur (*p*<0.001 for all) at the baseline compared to the participants who remained in the healthy-to-mild state (Supplementary Table 3). Similar findings were observed in men and women (*p*<0.001 for all) who worsened to severe sleep apnea at the follow-up. In addition, the average DesArea (*p*=0.002) and average DesDur (*p*=0.004) were significantly higher at baseline in women who worsened to moderate sleep apnea. Furthermore, avg. DesArea in men (*p*=0.012) and women (*p*=0.002) and avg. DesDur in women (*p*=0.014) were significantly higher in participants who worsened to severe sleep apnea. The only statistically significant difference between participants who worsened to moderate sleep apnea and participants who worsened to severe sleep apnea was observed in DesSev (*p*=0.035) in the group consisting of both sexes.

DesSev (*p*=0.025) and DesDur (*p*=0.036) parameter values were statistically significantly higher in participants with mild sleep apnea who underwent only baseline PSG compared to the participants who underwent both PSGs, although the difference in the absolute values was small (Supplementary Table 4).

The ODI, DesSev, and DesDur parameter values determined from automatic scorings were statistically significantly higher compared to the values determined from manual scorings (Supplementary Table 5). However, the correlations (ρ≥0.87) and agreements between the scores were excellent and median differences in parameter values were small (Supplementary Table 5, Supplementary Figure 1).

Supplementary Table 1. Demographic, anthropometric, and desaturation parameter values for men and women participants with mild sleep apnea (5≤ODI3%<15) at the baseline and after the mean follow-up time of 5.2 years.

|  | **Men** | | | **Women** | | |
| --- | --- | --- | --- | --- | --- | --- |
|  | Baseline | Follow-up | Change during the follow-up | Baseline | Follow-up | Change during the follow-up |
| **Number of patients (*n*)** |  | 393 |  |  | 589 |  |
| **Follow-up time (y)** |  | 5.2 (0.3) |  |  | 5.2 (0.2) |  |
| **Age (y)** | 61.0 (10.3) | 66.3 (10.2)* | 5.2 (0.5) | 63.1 (10.1)^†^ | 68.4 (10.0)* | 5.2 (0.5) |
| **BMI (kg/m^2^)** | 27.3 (3.7) | 27.6 (3.8)* | 0.3 (2.1) | 27.5 (4.8) | 27.7 (5.1)* | 0.1 (2.3) |
| **TST (h)** | 6.1 (0.9) | (6.2) (1.0) | 0.1 (1.1) | 6.4 (0.9)^†^ | 6.4 (1.2) | 0.1 (1.3) |
| **Supine time (%)** | 22.2 (0.0, 51.8) | 23.0 (8.0, 53.0) | 2.3 (36.4) | 22.3 (0.7, 58.5) | 30.0 (10.0, 57.5) | 2.7 (44.0) |
| **NC (cm)** | 39.8 (2.6) | 39.6 (2.8)* | -0.2 (2.2) | 34.7 (2.7)^†^ | 34.8 (2.8) | 0.0 (2.0)^†^ |
| **NC/H (%)** | 22.7 (1.5) | 22.8 (1.8) | 0.0 (1.3) | 21.6 (1.7)^†^ | 21.8 (1.8)* | 0.2 (1.3)^†^ |
| **REM (%)** | 20.6 (6.4) | 21.0 (6.8) | 0.4 (8.3) | 20.2 (6.7) | 20.7 (6.3) | 0.5 (8.0) |
| **Hypertension (*n*, %)** | 158 (40.2) | 192 (48.9) |  | 253 (43.0) | 306 (52.0) |  |
| **Diabetes (*n*, %)** | 25 (6.4) | n.a. |  | 27 (4.6) | n.a. |  |
| **CVD (*n*, %)** | 60 (15.3) | 85 (21.6) |  | 35 (5.9)^†^ | 63 (10.7) |  |
| **ODI (1/h)** | 9.6 (7.4, 11.7) | 15.3 (9.9, 21.7)* | 8.7 (13.3) | 8.9 (6.7, 11.6)^†^ | 14.1 (9.4, 20.2)* | 7.4 (11.0) |
| **DesSev (%)** | 0.20 (0.08) | 0.38 (0.29)* | 0.19 (0.27) | 0.18 (0.08)^†^ | 0.34 (0.27)* | 0.17 (0.25) |
| **DesDur (%)** | 8.3 (2.8) | 14.9 (10.0)* | 6.6 (9.4) | 7.6 (2.8)^†^ | 13.4 (8.4)* | 5.8 (7.9) |
| **avg. DesArea (s%)** | 72.2 (17.7) | 74.1 (19.3)* | 1.9 (19.7) | 68.2 (18.1)^†^ | 72.4 (19.9)* | 4.2 (18.4)^†^ |
| **avg. DesDur (s)** | 29.8 (5.7) | 29.1 (5.3)* | -0.7 (5.9) | 28.2 (5.8)^†^ | 28.6 (5.7) | 0.3 (6.3)^†^ |

Data are presented as means and standard deviations for normally distributed variables, as medians and interquartile ranges for non-normally distributed variables, and as *n* and percentages for categorical variables. Statistical significance (*p*<0.05) between baseline and follow-up measurements was determined with Wilcoxon signed-rank test (*). Mann-Whitney *U* and Chi-squared tests were used to compare baseline parameter values for continuous and categorical variables, respectively, and changes in parameter values during the follow-up between the sexes (†). BMI = body mass index, TST = total sleep time, NC = neck circumference, NC/H = neck circumference/height ratio, REM = rapid eye movement sleep, CVD = cardiovascular disease (consisting of heart failure, stroke, myocardial infarction, coronary artery bypass graft, and coronary angioplasty), ODI = oxygen desaturation index, DesSev = desaturation severity parameter, DesDur = desaturation duration parameter, avg. DesArea = average area of individual desaturation events, avg. DesDur = average duration of individual desaturation events, n.a. = not available.

Supplementary Table 2. Linear regression analyses for the estimation of mild sleep apnea (5≤ODI3%<15) progression based on the desaturation parameter values at the baseline.

|  |  | **Unadjusted** |  |  | **Adjusted^‡^** |  |
| --- | --- | --- | --- | --- | --- | --- |
|  | β | SD error | *p*-value | β | SD error | *p*-value |
| **ODI (1/h)** |  |  |  |  |  |  |
| **Men** | 1.088 | 0.669 | 0.105 | 0.676 | 0.659 | 0.305 |
| **Women** | -0.339 | 0.454 | 0.455 | -0.725 | 0.460 | 0.115 |
| **Both** | 0.257 | 0.382 | 0.501 | -0.180 | 0.387 | 0.642 |
| **DesSev (%)** |  |  |  |  |  |  |
| **Men** | 2.330 | 0.661 | <0.001 | 1.740 | 0.650 | 0.008 |
| **Women** | 0.549 | 0.454 | 0.227 | 0.099 | 0.457 | 0.828 |
| **Both** | 1.301 | 0.380 | 0.001 | 0.890 | 0.384 | 0.021 |
| **DesDur (%)** |  |  |  |  |  |  |
| **Men** | 1.650 | 0.666 | 0.014 | 1.058 | 0.659 | 0.109 |
| **Women** | 0.491 | 0.454 | 0.280 | -0.011 | 0.460 | 0.981 |
| **Both** | 1.021 | 0.381 | 0.008 | 0.564 | 0.386 | 0.145 |
| **avg. DesArea (s%)** |  |  |  |  |  |  |
| **Men** | 1.932 | 0.664 | 0.004 | 1.369 | 0.655 | 0.037 |
| **Women** | 1.452 | 0.450 | 0.001 | 1.059 | 0.451 | 0.019 |
| **Both** | 1.701 | 0.379 | <0.001 | 1.437 | 0.380 | <0.001 |
| **avg. DesDur (s)** |  |  |  |  |  |  |
| **Men** | 1.049 | 0.669 | 0.118 | 0.413 | 0.663 | 0.534 |
| **Women** | 1.609 | 0.450 | <0.001 | 1.214 | 0.450 | 0.007 |
| **Both** | 1.457 | 0.380 | <0.001 | 1.184 | 0.381 | 0.002 |

(^‡^) Adjusted for age, body mass index, change in body mass index during the follow-up, neck circumference/height ratio, the existence of hypertension, diabetes, and cardiovascular diseases (consisting of heart failure, stroke, myocardial infarction, coronary artery bypass graft, and coronary angioplasty), percentage of time slept in the supine position, percentage of time slept in rapid eye movement sleep, change in rapid eye movement sleep between the polysomnographic recordings, and follow-up time. β-values correspond to the expedited increase in ODI between the PSG recordings that are associated with one standard deviation change in desaturation parameter values at the baseline. Standard deviations, for men, women, and the group consisting of both sexes were: for ODI 2.7, 2.9, and 2.8, for DesSev 0.08, 0.08, and 0.08, for DesDur 2.8, 2.8, and 2.8, for the average DesArea 17.7, 18.1, and 18.0, and average DesDur 5.7, 5.8, and 5.8, respectively. ODI = oxygen desaturation index, DesSev = desaturation severity parameter, DesDur = desaturation duration parameter, average DesArea = average area of individual desaturation events, average DesDur = average duration of individual desaturation events.

Supplementary Table 3. Desaturation parameter values at the baseline in participants whose sleep apnea severity remained in healthy-to-mild state (ODI3%<15) and participants whose disease worsened to moderate (15≤ODI3%<30) or severe (ODI3%≥30) sleep apnea during the follow-up.

|  | **ODI3%<15** | **15≤ODI3%<30** | **ODI3%≥30** |
| --- | --- | --- | --- |
| **ODI (1/h)** |  |  |  |
| **Men** | 8.3 (6.4, 10.7) | 10.7 (8.9, 13.1)* | 10.8 (9.4, 12.6)* |
| **Women** | 8.1 (6.3, 10.7) | 9.9 (7.5, 12.4)* | 10.5 (8.1, 13.0)* |
| **Both** | 8.2 (6.3, 10.7) | 10.2 (7.8, 12.7)* | 10.7 (8.5, 12.7)* |
| **DesSev (%)** |  |  |  |
| **Men** | 0.17 (0.07) | 0.22 (0.07)* | 0.23 (0.08)* |
| **Women** | 0.16 (0.07) | 0.20 (0.09)* | 0.21 (0.08)* |
| **Both** | 0.16 (0.07) | 0.20 (0.08)* | 0.22 (0.08)*^#^ |
| **DesDur (%)** |  |  |  |
| **Men** | 7.3 (2.5) | 9.2 (2.6)* | 9.4 (2.8)* |
| **Women** | 6.9 (2.6) | 8.2 (2.9)* | 8.9 (2.9)* |
| **Both** | 7.0 (2.6) | 8.6 (2.8)* | 9.1 (2.9)* |
| **avg. DesArea (s%)** |  |  |  |
| **Men** | 70.3 (17.7) | 73.0 (17.1) | 76.7 (18.3)* |
| **Women** | 65.4 (16.3) | 70.9 (19.1)* | 73.6 (20.5)* |
| **Both** | 67.3 (17.0) | 71.8 (18.3)* | 75.0 (19.5)* |
| **avg. DesDur (s)** |  |  |  |
| **Men** | 29.3 (6.1) | 30.1 (5.4) | 30.5 (5.2) |
| **Women** | 27.4 (5.3) | 29.1 (6.0)* | 29.6 (6.5)* |
| **Both** | 28.1 (5.7) | 29.5 (5.8)* | 30.0 (5.9)* |

Data are presented as means and standard deviations for normally distributed variables and as medians and interquartile ranges for non-normally distributed variables. Mann-Whitney *U* test was used to determine the statistical significance (*p*<0.05) between participants who remained in healthy-to-mild (*n*_Men_=192, *n*_Women_=320) state and participants who worsened to moderate (*n*_Men_=147, *n*_Women_=204) or severe (n_Men_=54, *n*_Women_=65) sleep apnea (*), and between participants who worsened to moderate sleep apnea and participants who worsened to severe sleep apnea (^#^). ODI = oxygen desaturation index, DesSev = desaturation severity parameter, DesDur = desaturation duration parameter, avg. DesArea = average area of individual desaturation events, avg. DesDur = average duration of individual desaturation events.

Supplementary Table 4. Comparison between participants with mild sleep apnea (5≤ODI3%<15) at the baseline who participated only to the baseline PSG and the participants who participated also to the follow-up PSG.

|  | **Participants with only baseline PSG** | **Participants with both PSGs** | ***p*-value** |
| --- | --- | --- | --- |
| ***n* (men, women)** | 984 (403, 581) | 982 (393, 589) |  |
| **Age (y)** | 63.9 (11.1) | 62.3 (10.2) | 0.002 |
| **BMI (kg/m^2^)** | 27.1 (4.3) | 27.4 (4.4) | 0.052 |
| **Supine time (%)** | 21.8 (0.0, 54.0) | 22.2 (0.2, 54.8) | 0.397 |
| **NC (cm)** | 36.9 (3.9) | 36.8 (3.7) | 0.584 |
| **NC/H (%)** | 22.2 (1.9) | 22.0 (1.7) | 0.051 |
| **REM (%)** | 19.6 (6.8) | 20.3 (6.6) | 0.012 |
| **Hypertension (*n*, %)** | 553 (56.2) | 411 (41.9) | <0.001 |
| **Diabetes (*n*, %)** | 82 (8.3) | 52 (5.3) | 0.008 |
| **CVD (*n*, %)** | 129 (13.1) | 95 (9.7) | 0.017 |
| **ODI (1/h)** | 9.5 (7.2, 12.2) | 9.1 (7.0, 11.6) | 0.071 |
| **DesSev (%)** | 0.19 (0.08) | 0.18 (0.08) | 0.025 |
| **DesDur (%)** | 8.1 (2.9) | 7.8 (2.8) | 0.036 |
| **avg. DesArea (s%)** | 70.9 (18.6) | 69.8 (18.0) | 0.168 |
| **avg. DesDur (s)** | 29.2 (6.2) | 28.9 (5.8) | 0.336 |

Values are presented as means and standard deviations for normally distributed parameters, as medians and interquartile ranges for non-normally distributed parameters, and as *n* and percentages for categorical variables. Statistical comparison of observed differences between the populations was investigated with Mann-Whitney *U* test for continuous variables and with Chi-squared test for categorical variables. BMI = body mass index , NC = neck circumference, NC/H = neck circumference height ratio, REM = rapid eye movement sleep, CVD = cardiovascular diseases (consisting of myocardial infarction, heart failure, stroke, coronary angioplasty, and coronary artery bypass graft), ODI = oxygen desaturation index, DesSev = desaturation severity parameter, DesDur = desaturation duration parameter, avg. DesArea = average area of individual desaturation events, avg. DesDur = average duration of individual desaturation events.

Supplementary Table 5. Automatically and manually scored oxygen saturation signal-derived parameters were similar and strongly correlated. For this analysis, 30 oxygen saturation signals were randomly selected from the Sleep Heart Health Study dataset of 8444 available recordings.

|  | Automatic scorings | Manual scorings | Difference | Spearman correlation |
| --- | --- | --- | --- | --- |
| **ODI3% (1/h)** | 11.1 (5.4, 19.9) | 9.9 (5.2, 17.8)* | 0.7 (0.4, 1.6) | 1.00 |
| **DesSev3% (%)** | 0.20 (0.11, 0.53) | 0.18 (0.10, 0.49)* | 0.01 (0.00, 0.02) | 0.98 |
| **DesDur3% (%)** | 8.5 (5.2, 18.2) | 7.5 (4.5, 16.9)* | 1.0 (0.6, 1.8) | 0.98 |
| **avg. DesArea3% (s%)** | 69.6 (59.0, 86.1) | 71.4 (58.5, 91.6) | -1.5 (-3.2, 1.0) | 0.87 |
| **avg. DesDur3% (s%)** | 28.7 (23.0, 33.3) | 27.2 (23.0, 32.8) | 0.2 (-0.4, 0.9) | 0.92 |

Values are presented as medians (interquartile range). The statistical significance of the observed difference between the scorings was investigated with Wilcoxon signed-rank test (**, p*<0.05). ODI = oxygen desaturation index, DesSev = desaturation severity parameter, DesDur = desaturation duration parameter, avg. DesArea = average area of individual desaturation events, avg. DesDur = average duration of individual desaturation events.


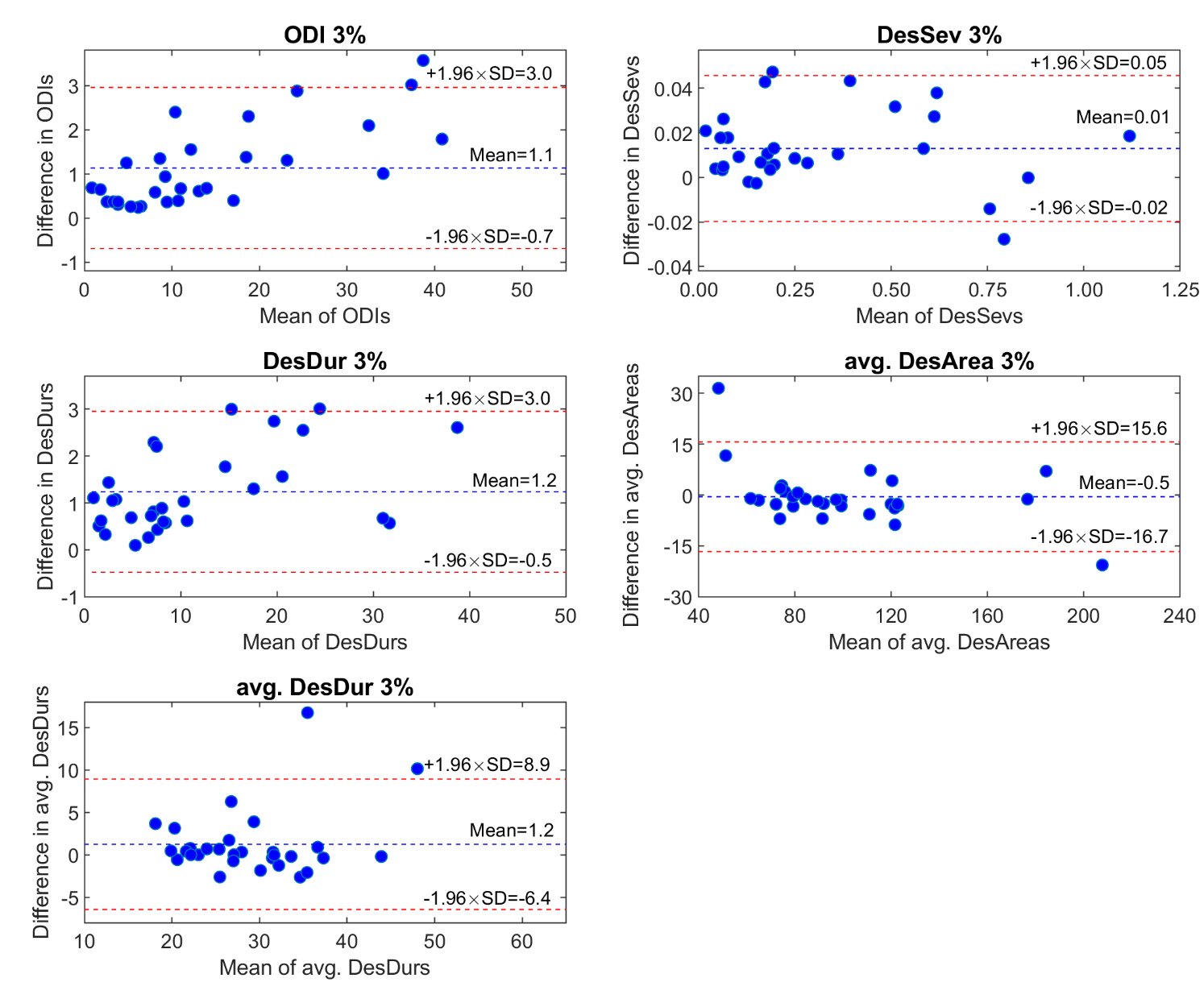
Supplementary Figure 1. Comparison of automatically and manually scored desaturation parameter values (3% criterion) of 30 randomly selected oxygen saturation signals using Bland-Altman plots. ODI, oxygen desaturation index; DesSev, desaturation severity parameter; DesDur, desaturation duration parameter; avg. DesArea, average area of individual desaturation events; avg. DesDur, average duration of individual desaturation events.
